# Comparative Analysis of Decellularization Methods for the Production of Decellularized Umbilical Cord Matrix

**DOI:** 10.3390/cimb46070455

**Published:** 2024-07-19

**Authors:** Yang Li, Yang Zhang, Guifeng Zhang

**Affiliations:** 1 National Key Laboratory of Biochemical Engineering, Institute of Process Engineering, Chinese Academy of Sciences, Beijing 100190, China; liyang2343@163.com (Y.L.); zhangyang21@ipe.ac.cn (Y.Z.); 2 School of Chemical and Engineering, University of Chinese Academy of Sciences, Beijing 100049, China

**Keywords:** decellularized extracellular matrix (dECM), decellularization technique, human umbilical cord tissue

## Abstract

The importance of decellularized extracellular matrix (dECM) as a natural biomaterial in tissue engineering and regenerative medicine is rapidly growing. The core objective of the decellularization process is to eliminate cellular components while maximizing the preservation of the ECM’s primary structure and components. Establishing a rapid, effective, and minimally destructive decellularization technique is essential for obtaining high-quality dECM to construct regenerative organs. This study focused on human umbilical cord tissue, designing different reagent combinations for decellularization protocols while maintaining a consistent processing time. The impact of these protocols on the decellularization efficiency of human umbilical cord tissue was evaluated. The results suggested that the composite decellularization strategy utilizing trypsin/EDTA + Triton X-100 + sodium deoxycholate was the optimal approach in this study for preparing decellularized human umbilical cord dECM. After 5 h of decellularization treatment, most cellular components were eliminated, confirmed through dsDNA quantitative detection, hematoxylin and eosin (HE) staining, and DAPI staining. Meanwhile, Masson staining, periodic acid-silver methenamine (PASM) staining, periodic acid-Schiff (PAS) staining, and immunofluorescent tissue section staining results revealed that the decellularized scaffold retained extracellular matrix components, including collagen and glycosaminoglycans (GAGs). Compared to native umbilical cord tissue, electron microscopy results demonstrated that the microstructure of the extracellular matrix was well preserved after decellularization. Furthermore, Fourier-transform infrared spectroscopy (FTIR) findings indicated that the decellularization process successfully retained the main functional group structures of extracellular matrix (ECM) components. The quantitative analysis of collagen, elastin, and GAG content validated the advantages of this decellularization process in preserving and purifying ECM components. Additionally, it was confirmed that this decellularized matrix exhibited no cytotoxicity in vitro. This study achieved short-term decellularization preparation for umbilical cord tissue through a combined decellularization strategy.

## 1. Introduction

Tissue engineering and regenerative medicine are of great significance to modern medicine and human health. They offer the possibility of regenerating damaged tissues and organs, addressing challenges that traditional medicine struggles with, such as the shortage of donor organs and immune rejection. By combining a patient’s own cells with biomaterials to cultivate new tissues or organs, the risk of immune rejection can be reduced, and customized treatments can be provided according to individual needs. This approach holds tremendous potential for future development. Decellularized extracellular matrix (dECM) is a complex secreted by cells into the extracellular space and constitutes an important part of the cellular microenvironment [1,2,3]. dECM is a natural biomaterial created by removing cellular components from tissue while retaining the extracellular matrix, utilizing various chemical, physical, and biological methods [4,5,6]. During decellularization, most cells and immunogenic molecules are eliminated, while a significant proportion of structural proteins such as collagen, elastin, fibronectin, and macromolecules including proteoglycans and glycosaminoglycans (GAGs) remain intact [7]. The application of dECM in the field of regenerative medicine has garnered widespread attention. This material not only possesses the biocompatibility of natural tissues/organs but also minimizes the risk of immune rejection by removing cellular and nuclear components. This advantage makes decellularized matrix materials an ideal choice for creating tissue-engineered artificial organs to replace damaged or dysfunctional tissues. Furthermore, the unique strength of decellularized matrix materials lies in their ability to mimic the microenvironment of natural tissues and exhibit bio-inductive functionality. The extracellular matrix (ECM) components and structure contained within the decellularized matrix material can provide cells with a growth microenvironment similar to natural tissues, encompassing essential biological signals and physical support. This aids cells in achieving directed tissue culture and tissue engineering reconstruction [8]. Recently, researchers have developed diverse application forms, including 3D-printed bio-ink [9,10], injectable hydrogels [11,12], and electrospun scaffolds [13], based on decellularized matrix materials. This has significantly broadened the range of applications for decellularized matrix as a tissue engineering product.

Theoretically, any organ or tissue rich in ECM has the potential to serve as a source of decellularized matrix. Currently, various tissues and organs, such as skin, heart, nerves, liver, kidney, iliotibial band, bladder, and cornea, are being used to construct decellularized matrices [14,15,16,17,18,19,20,21]. Given the limited clinical resources, the organs and tissues employed for constructing these matrices often originate from diverse species, such as pigs [22,23,24]. Umbilical cord tissue, as a regenerative medicine material, possesses inherent advantages over other decellularized matrix sources. Being a byproduct of childbirth, it is abundant and ethically acceptable, with ample clinical availability. Additionally, it exhibits lower immunogenicity compared to heterologous tissues, rendering it suitable for a wider range of treatment scenarios. Moreover, umbilical cord-derived tissue engineering materials are more ethically favorable than sources like embryonic stem cells. These advantages make the umbilical cord an ideal biomaterial source. The ECM components of the umbilical cord, comprising collagen, elastin, proteoglycans and GAGs, are highly biocompatible and supportive of cell growth and function. This makes it an ideal substrate for diverse biomedical applications, where it fosters cell adhesion, migration, proliferation, and differentiation [25,26]. By eliminating cellular components, decellularization significantly minimizes the risk of immune rejection when the umbilical cord matrix is implanted or used as a scaffold in the body. This is pivotal for the success of cell-based therapies and tissue engineering.

Current research on umbilical cord acellular matrix materials primarily focuses on the decellularization of Wharton’s jelly [27,28] and the utilization of decellularized arteries [29] or veins [30] as scaffolds for vascular grafts, nerve conduits, and other applications. However, there have been relatively few reports published on the decellularization of the entire umbilical cord tissue. Developing a comprehensive umbilical cord decellularization process is highly significant. This ECM serves as a natural scaffold that can be used in various biomedical applications, including tissue engineering, wound healing, and regenerative medicine. Furthermore, it is crucial to develop a rapid, efficient, and minimally invasive decellularization technique that produces high-quality dECM for regenerative organ construction. This study specifically aims to devise a decellularization protocol for whole umbilical cord tissue, grounded on the hypothesis that a combined decellularization procedure can effectively yield a decellularized and bioactive umbilical cord matrix. Furthermore, through various characterization methods, we compare and evaluate the decellularization efficacy of different reagent combinations on the tissue, as well as their ability to consistently preserve ECM components over time. The ultimate goal is to identify the most efficient method for preparing a decellularized umbilical cord matrix. Additionally, we delve into the potential mechanisms that may explain the observed patterns, giving depth to the significance of our findings. This research serves as a theoretical foundation for applications in tissue engineering and regenerative medicine practices.

## 2. Materials and Methods

### 2.1. Collection of Umbilical Cord Tissue

The umbilical cord (UC) was collected from discarded clinical materials, and explicit permission was granted by the mothers involved. The review board and ethics committee of Zhujiang Hospital of Southern Medical University have scrutinized and sanctioned the study protocols (approval number: 2022-KY-003-01). The umbilical cord was sourced from mothers who met the criteria of being under 28 years old at the time of childbirth and were confirmed to be free from any infectious diseases, including hepatitis B virus and HIV. Immediately after detachment from the body, the umbilical cord was placed into a centrifuge tube containing pre-cooled, sterile PBS (4 °C). It was subsequently placed in an ice box and transported to the laboratory, where it underwent a simple rinse prior to being frozen and preserved in a −80 °C freezer.

### 2.2. Preparation of Umbilical Cord dECM

After the umbilical cord tissue was removed from the freezer, it was thawed in a 37 °C water bath. The umbilical cord tissue was sliced longitudinally into thin sections (5 cm × 2 cm × 0.5 cm). These sections were placed in ultrapure water and shake-rinsed three times on a shaking bed for 10 min each time. Seven decellularization protocols had been established, each maintaining a uniform processing time. The protocols were performed as shown in Table 1. The reagents employed in these procedures consist of trypsin-EDTA solution from Thermo Fisher Scientific (Waltham, MA, USA), Triton X-100 by Macklin Inc. (Shanghai, China), and Sodium Deoxycholate (SD), also sourced from Macklin Inc. (Shanghai, China).

The tissue was subsequently washed thoroughly with deionized water to eliminate any remaining reagent residues. A vacuum freeze-dryer (LGJ-20F, SONG YUAN FREEZE DRYER, Beijing, China) was utilized to freeze-dry the umbilical cord tissue for a duration of 48 h, resulting in a dehydrated, freeze-dried, and decellularized matrix of human umbilical cord.

### 2.3. Analysis of Umbilical Cord dECM

#### 2.3.1. Quantitative Analysis of dsDNA

To facilitate further detection, the freeze-dried matrices were sliced and inserted into a grinding device (LUKYM-I, Luka, Guangzhou, China). They were then pulverized at a frequency of 55 HZ for 180 s, eventually producing a delicate powder composed of human umbilical cord decellularized matrix.

Amounts of 5 mg of natural umbilical cord tissue powder and human umbilical cord decellularized matrix powder, derived from various decellularization protocols, were separately weighed out. DNA was extracted from the decellularized matrix in strict accordance with the instructions provided in the tissue genomic DNA extraction kit (Tiangen Biotech (Beijing) Co., Ltd., Beijing, China). A nanodrop micro nucleic acid analyzer (Thermo Fisher Scientific, Waltham, MA, USA) was then utilized to ascertain the dsDNA content present in the extracted DNA solution. Finally, the obtained value was converted to represent the dsDNA content per milligram of dry tissue powder, ensuring accurate quantification.

#### 2.3.2. Histological Analysis

For histological analyses, both the native umbilical cord tissue and its decellularized counterpart underwent a crucial preservation step through fixation using 4% paraformaldehyde (Macklin Inc., Shanghai, China). This process stabilized the tissue structure, preserving its histological features for detailed examination. Following fixation, the tissues were paraffin-embedded, a technique that allowed for thin sectioning and permanent mounting of the samples.

Using a precision microtome (RM216, Leica, Bensheim, Germany), thin tissue sections were carefully prepared. These sections were then mounted onto slides, ready for staining and microscopic observation. After deparaffinization, a series of histological stains were applied to highlight different tissue components. Hematoxylin and eosin (HE) (Beyotime Biotechnology, Shanghai, China) staining offered a general overview of tissue architecture, while specialized stains like periodic acid-silver methenamine (PASM) (LEAGENE Biotechnology, Beijing, China), periodic acid-Schiff (PAS) (Beyotime Biotechnology, Shanghai, China), and Masson (Beyotime Biotechnology, Shanghai, China) provided insights into specific tissue structures and components. After acquiring the images, the integrated optical density of each image was further analyzed using the ImageJ software package (Version 1.51j8).

To delve deeper into the location of decellularized matrix elements within these tissue sections, immunofluorescence staining was employed on the paraffin-embedded samples. This highly sensitive technique utilized polyclonal antibodies targeting collagen I and elastin (both antibodies from Proteintech Group, Inc., Chicago, IL, USA) to specifically label these matrix components. Once the primary antibodies had bound to their targets, secondary antibodies were introduced, followed by the addition of 4′,6-diamidino-2-phenylindole (DAPI) (Beyotime Biotechnology, Shanghai, China) to stain the cell nuclei. This comprehensive staining protocol facilitated a detailed analysis of the tissue samples, revealing the intricate network of the extracellular matrix and its relationship to cellular components. Meanwhile, the integrated optical density of each image was also further analyzed using the ImageJ software package.

#### 2.3.3. Scanning Electron Microscopy (SEM) Analysis

The surface micromorphology of the natural freeze-dried umbilical cord, as well as the freeze-dried umbilical cord that underwent various decellularization treatments, was examined using scanning electron microscopy (SEM, JEOL JSM-6700F, Akishima, Japan). The samples were trimmed to suitable sizes and affixed to the microscope stage with conductive adhesive. After creating a vacuum inside the ion beam sputtering instrument, gold was sprayed onto the samples (using a spray current of 30 mA and a spray duration of 120 s) to enhance the sample signal and minimize electrostatic effects. Once gold spraying was complete, the samples were removed. Subsequently, the electron beam was activated to scan the surface of the samples. The appropriate working distance and magnification were selected based on our requirements, and the captured image data were analyzed and documented to gain insights into the microstructure and surface features of the samples.

#### 2.3.4. Fourier-Transform Infrared Spectroscopy

Utilizing a NICOLET iS50 FT-IR spectrometer (Thermo Fisher Scientific, Waltham, MA, USA), the Fourier-transform infrared (FTIR) spectra of both the untreated freeze-dried umbilical cord and that subjected to different decellularization treatments were obtained. The spectrometer was set in transmittivity mode, scanning the spectral range from 1200 cm^−1^ to 3600 cm^−1^ at a resolution of 4 cm^−1^. For precision, each spectrum was derived by averaging data from 32 scans. The peak area of the characteristic absorption peaks in FTIR was calculated using the OriginPro 8.5 software.

#### 2.3.5. Quantitative Analysis

Appropriate amounts of natural freeze-dried umbilical cord powder and freeze-dried umbilical cord powder that had undergone different decellularization treatments were weighed and placed into a 1.5 mL centrifuge tube, along with 750 μL of 0.25 mol/L oxalic acid solution (Tianjin Damao Chemical Reagents Factory, Tianjin, China). The mixture was then heated in a 100 °C water bath for 1 h and subsequently centrifuged at a high speed of 10,000 r/min for 10 min. The supernatant was retained, and the elastin content in the ECM was detected in accordance with the instructions provided in the Fastin^TM^ elastin Assay (QuickZime Biosciences, Leiden, The Netherlands) and the soluble collagen was detected in accordance with the instructions provided in the Sircol^TM^ Soluble Collagen Assay (Biocolor Life Sciences, Carrickfergus, UK).

The tissue powders, as mentioned above, were weighed and mixed with 0.1 mg/mL papain solution (prepared in 0.2 mol/L sodium phosphate buffer at pH 6.4). The mixture was then heated in a 65 °C water bath for enzymatic hydrolysis lasting 3 h, followed by high-speed centrifugation at 10,000 r/min for 10 min. The supernatant was subsequently collected, and the elastin content in the ECM was detected in accordance with the instructions provided in the BlyscanTM sGAG assay kit (Biocolor Life Sciences, Carrickfergus, UK).

#### 2.3.6. In Vitro Cytotoxicity Analysis

In this experiment, human liver progenitor cells (HepaRG cells) were chosen as the test cells, and HepaRG cell complete medium was used as the extraction medium. HepaRG cells were purchased from Biopredic International (Rennes, France) and were cultured in William’s E Medium (Thermo Fisher Scientific, Waltham, MA, USA) supplemented with Metabolism Medium Supplement (Thermo Fisher Scientific, Waltham, MA, USA) at 37 °C in a CO_2_ incubator (MCO-20AIC, SANYO, Osaka, Japan). The decellularized matrix powders of the Try + SD, Tri + SD, and Try + Tri + SD groups were combined with the extraction medium at a ratio of 0.2 g/mL and extracted for 24 h at a temperature of 37 ± 1 °C. The supernatant of the extraction fluid was then collected for subsequent tests. The negative control group consisted of sterile high-density polypropylene (PP) mixed with the extraction medium at a ratio of 3 cm^2^/mL. The positive control group used complete medium with 10% dimethyl sulfoxide (DMSO) (Biosharp, Hefei, China). HepaRG cells in the logarithmic growth phase were seeded at a concentration of 1 × 10^5^ cells/mL in a 96-well plate. After 24 h of cell adhesion, the liquid in the wells was removed and 100 µL of the extraction fluid from the Try + SD, Tri + SD, Try + Tri + SD experimental groups, positive control group, negative control group, and fresh cell culture medium as a blank control group were added, respectively. The cells were then incubated in a CO_2_ incubator for 24 h. The morphological changes in the cells were observed and recorded under an inverted microscope. The liquid in the wells was aspirated, and 50 µL of a 1 mg/mL thiazolyl blue (MTT) solution (Biosharp, Hefei, China) was added to each well. After 2 h of incubation, the liquid in the wells was aspirated again and 100 µL of acidified isopropanol was added. After shaking for 10 min, the absorbance was measured at a wavelength of 570 nm. The survival rate of the cells was calculated according to the following formula:

$$\mathrm{Survival}\;\mathrm{rate}\;(\%)=\frac{100\times {\mathrm{OD}}_{570e}}{{\mathrm{OD}}_{570b}}$$

where OD_570e_ is the average optical density of the extract from the sample group, and OD_570b_ is the average optical density of the blank control group.

### 2.4. Statistical Analysis

All of the quantitative data were presented as mean ± standard deviation. Statistical analyses were performed using one-way ANOVA, followed by a Tukey HSD post hoc test. The *p*-values less than 0.05 were considered statistically significant.

## 3. Results

### 3.1. Preliminary Screening of Decellularization Protocols for Umbilical Cord dECM

In the initial stage, we tested the tissue DNA residue from seven distinct umbilical cord decellularization protocols. The preliminary screening criterion was derived from the literature, mandating that the dsDNA content should be reduced to less than 50 ng/mg of tissue dry weight [31]. This ensures the adequate removal of cellular and nuclear materials, thereby minimizing potential immune reactions.

**Figure 1:**
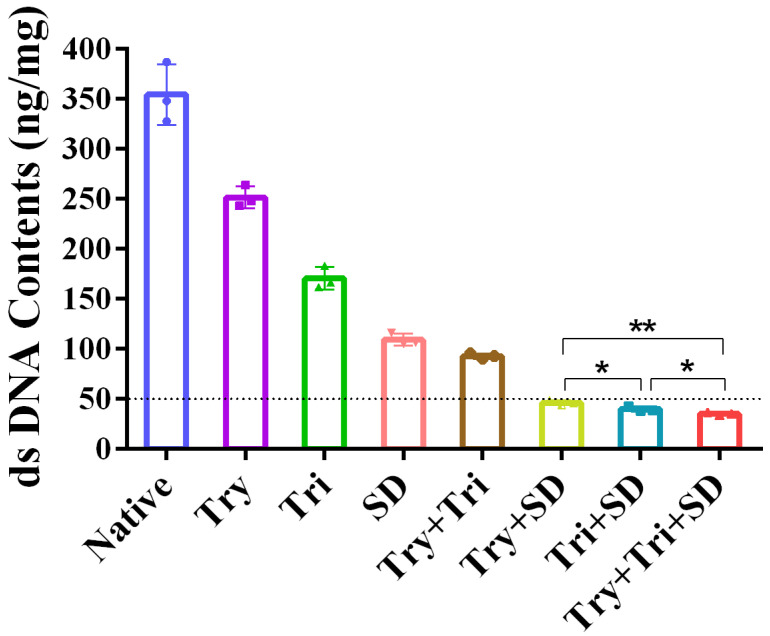
The dsDNA contents for each decellularization protocol. Data are mean ± SD; *n* = 3; *: *p* < 0.05; **: *p* < 0.01.

After testing, it was observed that compared to natural umbilical cord tissue, the dsDNA content of the decellularized tissue decreased to varying degrees within each treatment group (Figure 1). Notably, the Try + SD group (using trypsin and SD as chemical treatment reagents), the Tri + SD group (employing Triton X-100 + SD), and the Try + Tri + SD group (utilizing trypsin, Triton X-100, and SD) successfully achieved the dsDNA content standard of less than 50 ng/mg of tissue dry weight. Conversely, other treatment groups, including those involving single treatments with these chemical reagents and the Try + Tri group (using trypsin and Triton X-100), had a dsDNA content exceeding 50 ng/mg of tissue dry weight. The dsDNA residues for the Try + SD, Tri + SD, and Try + Tri + SD groups were measured at 46.25 ± 1.88 ng/mg, 40.02 ± 2.03 ng/mg, and 35.24 ± 1.18 ng/mg, respectively, indicating dsDNA removal efficiencies of 86.95%, 88.71%, and 90.06%. Among these, the Triton X-100 + SD group exhibited the most effective DNA removal. Furthermore, a statistically significant difference was observed between the Try + SD and Tri + SD groups (*p* < 0.05).

Based on the above results, we eliminated the Try group, Tri group, SD group, and Try + Tri group through preliminary dsDNA screening. Meanwhile, we retained the Try + SD group, Tri + SD group, and Try + Tri + SD group as candidate protocols for decellularized umbilical cord tissue to undergo further validation, with the ultimate goal of selecting the best protocol. Simultaneously, we observed that given the same treatment duration, the decellularization effect achieved by a single chemical component is less effective than that achieved by the combined chemical components. Furthermore, the finding that the residual dsDNA in the Try + SD group is greater than that in the Tri + SD group suggests that SD exhibits a stronger elution effect compared to Triton X-100.

### 3.2. Histological Analysis: Further Analysis of Cell Residues and Tissue Component Retention

#### 3.2.1. Immunohistochemical Staining

**Figure 2:**
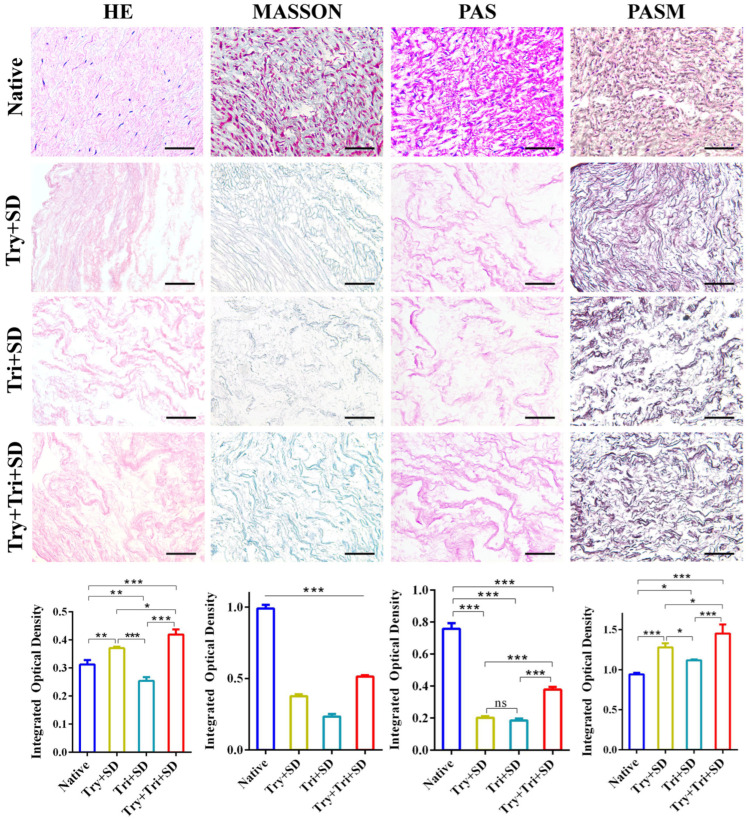
Immunohistochemical staining for each decellularization protocol. Scale bar: 50 μm. Data are mean ± SD; *n* = 3; ns: not significant; *: *p* < 0.05; **: *p* < 0.01; ***: *p* < 0.001.

The immunohistochemical staining results of umbilical cord tissue, both before and after decellularization, are displayed in Figure 2. The HE staining results showed numerous cellular structures in the untreated tissue, with nuclei distinctly stained blue, indicating a rich abundance of cellular components. However, after decellularization in the Try + SD, Tri + SD, and Try + Tri + SD groups, no obvious cellular remnants were visible, and the extracellular matrix appeared bright red. Notably, the Try + Tri + SD group not only exhibited a more pronounced color and denser tissue distribution compared to the Try + SD and Tri + SD groups, but also demonstrated a structure that was more similar to that of native tissue.

Masson’s staining revealed that collagen fibers in native umbilical cord tissue were stained blue with bright green, the cytoplasm was stained red, and the nuclei were stained purple-black. Following decellularization, no purple-black nuclear structures were observed in any of the three treatment groups; only the blue color remained, indicating the retention of a significant amount of collagen fiber components. Notably, the Try + Tri + SD group exhibited a more vivid color and denser tissue distribution.

Both PAS and PASM staining confirmed the absence of nuclear staining in the extracellular matrix post-decellularization, signifying the preservation of collagen, polysaccharides, and other matrix components. Consistently, the Try + Tri + SD group demonstrated superior staining effects compared to the other two groups.

Immunohistochemical staining further corroborated the effectiveness of decellularization, with a reduction in dsDNA content to less than 50 ng/mg of tissue dry weight serving as a preliminary indicator. No cellular remnants were detected in any of the decellularized groups. The Try + Tri + SD group displayed extracellular matrix components with a distribution and orientation that closely resembled that of natural umbilical cord tissue, suggesting minimal damage to the extracellular matrix in this group. In contrast, the Tri + SD group exhibited the most significant damage, resulting in a loosely organized tissue structure.

The integrated optical density for the above-mentioned staining methods consistently reveal that, in comparison to the Try + SD group and the Tri + SD group, the Try + Tri + SD group exhibited significantly higher optical density values. Additionally, the results of HE and PASM staining were notably superior to those of native tissues, likely due to the removal of impurities that interfere with staining during the decellularization process, ultimately enhancing the intensity of staining.

#### 3.2.2. Immunofluorescence Staining

**Figure 3:**
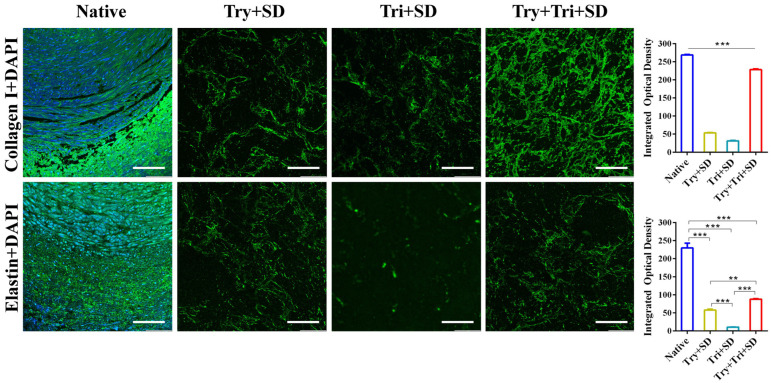
Immunofluorescence staining for each decellularization protocol. Scale bar: 100 μm. Data are mean ± SD; *n* = 3; **: *p* < 0.01; ***: *p* < 0.001.

Figure 3 displays the immunohistochemical staining results of umbilical cord tissues subjected to various decellularization treatments using specific protein antibodies. Type I collagen is the predominantly expressed collagen type within the extracellular matrix, while elastin is the primary constituent of elastic fibers. Together, collagen and elastin form the fibrous scaffold within the extracellular matrix. By comparing the staining results of type I collagen and elastin, we can evaluate the degree of tissue structure impairment caused by different decellularization techniques. The results show that decellularized umbilical cord tissues exhibit a noticeable degree of green fluorescence in both type I collagen and elastin expression. Notably, the Try + Tri + SD group shows stronger green fluorescence compared to the Try + SD and Tri + SD groups. In contrast to the Try + SD group, the Tri + SD group demonstrates weaker fluorescence expression, consistent with the findings in Section 3.2.1. Furthermore, through DAPI immunostaining, no discernible blue nuclear staining is observed in the decellularized treatment group when viewed under a fluorescence microscope. This observation underscores the significant elution effects of these three decellularization methods on the cells within the umbilical cord tissue, effectively removing cellular components. The most pronounced fluorescence expression of type I collagen is evident in the Try + Tri + SD group, indicating that the Try + Tri + SD decellularization approach has the least impact on type I collagen. Regarding elastin expression, the Try + SD group is comparable to the Try + Tri + SD group, while the expression in the Tri + SD group is comparatively weaker. This suggests a potentially detrimental effect of the Tri + SD decellularization scheme on elastin.

A further analysis of the integrated optical density of the obtained images revealed that while there were significant differences compared to native umbilical cord tissue, the Try + Tri + SD group exhibited higher optical density values in the expression of type I collagen and elastin when compared to the Try + SD group and the Tri + SD group.

#### 3.2.3. SEM Analysis

**Figure 4:**
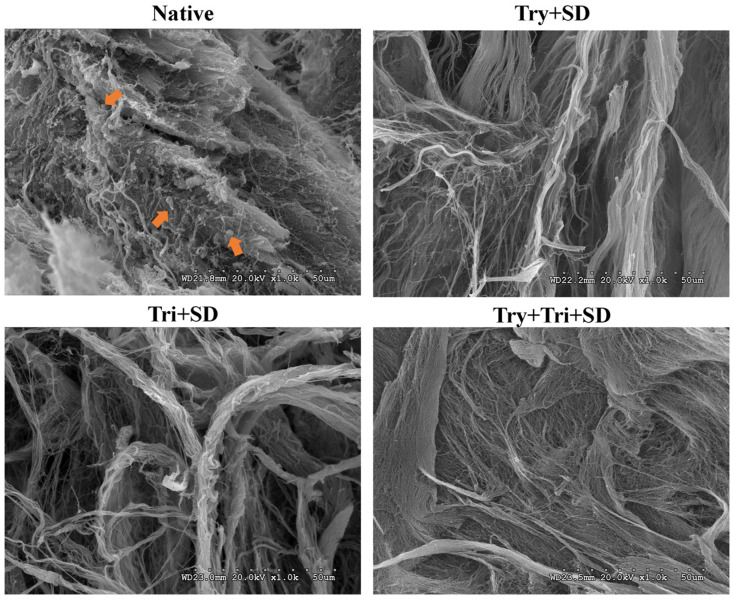
SEM analysis for each decellularization protocol. Scale bar: 50 μm. Orange arrows: cell structure.

The ultrastructure of the tissue before and after decellularization was further examined using scanning electron microscopy (Figure 4). The significant spherical cell structures on the surface and interior of fresh umbilical cord tissue, as indicated by the arrows, were absent in the Try + SD, Tri + SD, and Try + Tri + SD groups following decellularization treatment. Additionally, the tissue image of the Try + Tri + SD group exhibited a smooth, dense, and flat surface morphology. In contrast, the tissue fibers of the Try + SD and Tri + SD groups were arranged in a disorderly fashion, exhibiting significant fiber breakage and damage. These results suggest that all three elution treatment schemes are effective in removing cells, though the Try + SD and Tri + SD groups have a destructive effect on the ultrastructure of the tissue, while the Try + Tri + SD group does not cause significant tissue morphology damage.

#### 3.2.4. Fourier-Transform Infrared Spectroscopy

**Figure 5:**
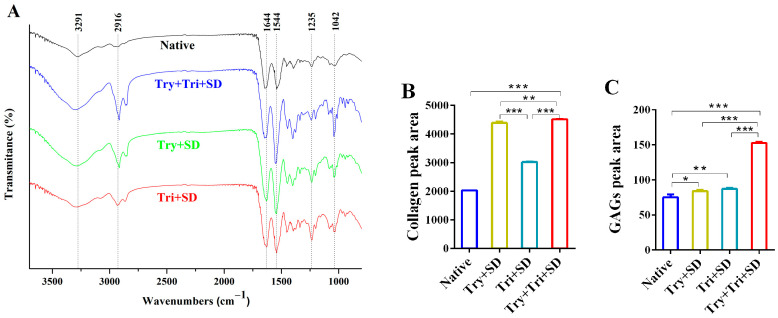
FTIR spectra for each decellularization protocol. (**A**) FTIR spectra. (**B**) Feature peak area of collagen spectra. (**C**) Feature peak area of GAG spectra. Data are mean ± SD; *n* = 3; *: *p* < 0.05; **: *p* < 0.01 ***: *p* < 0.001.

The FTIR analysis was performed on both native and decellularized umbilical cord tissues. The results indicated that the dECM of the umbilical cord exhibited characteristic absorption peaks of collagen at specific wavenumbers. Specifically, these peaks were located at 3291 cm^−1^, 2916 cm^−1^, 1644 cm^−1^, 1544 cm^−1^, and 1235 cm^−1^, corresponding to Amide A, Amide B, Amide I, Amide II, and Amide III, respectively. Studies have demonstrated that in FTIR, a peak ratio of approximately 1.0 between the amide III band and the peak within the 1400–1454 cm^−1^ range signifies an intact triple-helical structure of collagen [32]. As depicted in Figure 5, the collagen amide III band for both the native umbilical cord and the three decellularized treatment groups exhibits a wave number of 1235 cm^−1^, with a corresponding peak ratio of approximately 1.0 with the 1446 cm^−1^ band. This finding underscores that the collagen in these materials retains a relatively intact triple-helical structure, indicative of its preserved biological activity to a certain degree. Additionally, a distinct absorption peak indicative of GAGs appeared at 1042 cm^−1^ (Figure 5A) [33]. This suggests that the decellularization process effectively preserved the primary functional group structures of the ECM components.

In comparison to native umbilical cord tissue, the characteristic peaks in the decellularized umbilical cord tissue showed increased intensities and more distinct shapes. Further calculations were conducted on the peak areas of collagen (Figure 5B) and GAGs (Figure 5C) in different experimental groups. Specifically, the peak areas of collagen and GAG characteristic absorption peaks in the Try + SD, Tri + SD, and Try + Tri + SD groups were all significantly increased compared to natural umbilical cord tissue. Among these groups, the Try + Tri + SD group exhibited the largest peak area of characteristic absorption peaks, which was statistically different from those in the Try + SD and Tri + SD groups. The occurrence of this result may be attributed to the removal of cells and other impurities, which in turn enhances the functional group signals of the functional protein components on the surface of the decellularized matrix. This enhancement of signal strength may contribute to an improvement in the functionality of the decellularized matrix.

#### 3.2.5. Quantitative Analysis of Collagen, Elastin, and GAGs

**Figure 6:**
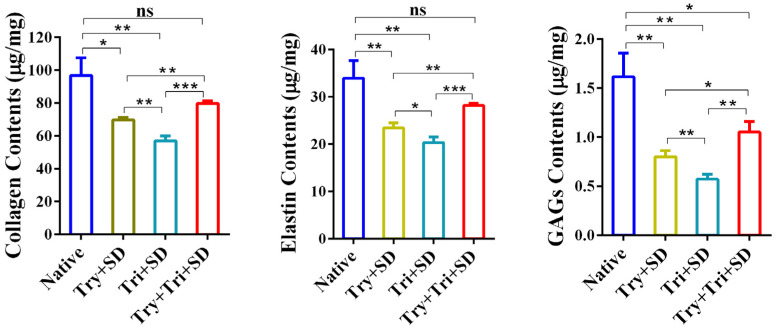
Quantitative analysis of collagen, elastin, and GAGs for each decellularization protocol. Data are mean ± SD; *n* = 3; ns: not significant; *: *p* < 0.05; **: *p* < 0.01; ***: *p* < 0.001.

Further examination of the collagen, elastin, and GAG content in natural and decellularized umbilical cord tissue was conducted. The results showed that compared to natural umbilical cord tissue, the content of collagen, elastin, and GAGs in decellularized umbilical cord tissue all displayed a decreasing trend (Figure 6). Specifically, the Tri + SD group showed the most significant decrease, with collagen, elastin, and GAG contents of 57.07 ± 2.36 μg/mg, 1.05 ± 0.08 μg/mg, and 0.57 ± 0.04 μg/mg, respectively. This indicates that this regimen causes the most severe disruption to the extracellular matrix. Meanwhile, the Try + Tri + SD group had significantly higher levels of collagen, elastin, and GAGs than the Try + SD and Tri + SD groups, specifically at 74.11 ± 7.23 μg/mg, 27.16 ± 1.23 μg/mg, and 1.05 ± 0.09 μg/mg, respectively (*p* < 0.05). The Try + Tri + SD group demonstrated a decline in the levels of GAGs, collagen, and elastin when compared to native umbilical cord tissue. However, only the reduction in GAG content was statistically significant (*p* < 0.05), while the changes in collagen and elastin levels did not reach statistical significance (*p* > 0.05). These results suggest that the Try + Tri + SD group’s decellularization process is gentler compared to the Try + SD and Tri + SD groups, resulting in minimal disruption to the ECM components of umbilical cord tissue.

#### 3.2.6. In Vitro Cytotoxicity Analysis

**Figure 7:**
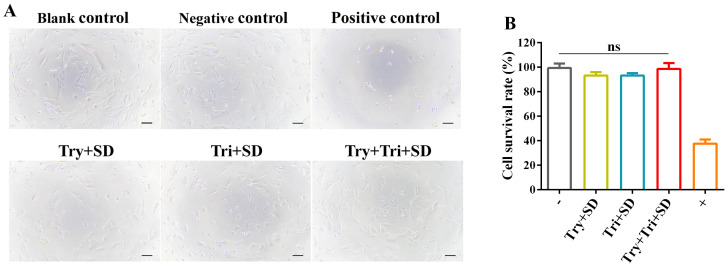
Cytotoxicity (in vitro) of each decellularization protocol. (**A**) Cell morphology observation. Scale bar: 100 μm. (**B**) Cell survival rate. Data are mean ± SD; *n* = 3; ns: not significant.

In the cultures treated with the extracts of the Try + SD, Tri + SD, and Try + Tri + SD groups, HepaRG cells exhibited normal growth status, characterized by a plump morphology, where most cells appeared as polygons or fusiform shapes. These morphological features closely resembled those of the cells in the negative control group and the blank control group. In contrast, the cells in the positive control group displayed significantly poorer growth, exhibiting atrophic cell morphology and most cells failing to adhere to the substrate (Figure 7A).

The results of cell viability calculations are presented in Figure 7B. Specifically, the cell viability rates were as follows: Try + SD, 93.29 ± 2.22%; Tri + SD, 93.27 ± 1.52%; Try + Tri + SD, 98.63 ± 3.84%; negative control, 99.39 ± 2.96%; positive control, 37.64 ± 2.66%. No statistically significant differences were observed between the Try + SD, Tri + SD, and Try + Tri + SD groups and the negative control group. These findings suggest that the extract cultures from the Try + SD, Tri + SD, and Try + Tri + SD groups are non-cytotoxic.

## 4. Discussion

Decellularization typically involves a series of steps designed to remove cellular components from tissues thoroughly, while preserving the integrity and functionality of the ECM. Distinct decellularization processes may be required for different tissues and organs, often necessitating a combination of methods to achieve the optimal decellularization. Currently, the most common decellularization methods include physical, biological, and chemical approaches. Physical methods, as a crucial component of decellularization, primarily rely on various techniques to disrupt cell membranes. These include ultrasonic treatment, repeated freezing and thawing [34], ultra-high pressure processing [35], and mechanical agitation. Although physical methods play a pivotal role in the decellularization process, they often fall short of completely removing cellular components from tissues and thus need to be supplemented with other methods. Biological methods constitute another approach to decellularization, involving the use of enzymes like trypsin, lipase, and nuclease. Trypsin, a proteolytic enzyme, targets the peptide bonds adjacent to the carboxylic groups of arginine and lysine residues within the peptide chain. Cleaving these peptide bonds can induce the lysis of cell adhesion proteins, ultimately leading to the separation of cells from the ECM. Importantly, trypsin itself is not cytotoxic, which is crucial for the development of medical materials. However, due to its potentially destructive nature, the treatment time and temperature must be carefully controlled to prevent damage to the ECM. As such, trypsin is often used in conjunction with other decellularization reagents. Yang et al. have found that trypsin may be essential as the initial step in a tissue decellularization protocol, particularly for completely removing cell nuclei from dense tissues [36]. Under the influence of trypsin, cells gradually detach from the ECM, facilitating subsequent decellularization steps. Chemical methods are also widely employed in decellularization. Their core principle lies in altering the permeability of cell membranes using specific chemical reagents. As the permeability of the cell membrane changes, cells gradually swell and eventually rupture, thereby achieving the desired decellularization. Commonly used chemical reagents include ionic detergents like SD and sodium dodecyl sulfate (SDS), as well as the non-ionic detergent Triton X-100. The structure and concentration of these reagents can impact the proteins within the ECM, potentially resulting in the partial damage or loss of ECM components [37]. Previous studies have indicated that non-ionic detergents are better suited for the decellularization of thinner tissues compared to ionic detergents. They inflict lesser damage on tissue microstructure and components such as proteins and GAGs [38]. On the other hand, ionic detergents, while highly efficacious in decellularization, may harm collagen, disrupt tissue microstructure, and cause irreversible protein denaturation, making them more appropriate for the decellularization of dense tissues.

In order to improve the efficiency of decellularization, researchers often combine multiple methods such as physical, chemical, and enzymatic techniques. This integrated processing strategy leverages the strengths of various methods while compensating for their respective weaknesses, achieving a more efficient and thorough decellularization effect. Faiza Ramzan et al. adopted a strategy of treating human umbilical cord tissue with 0.05% *w*/*v* trypsin for 2 h followed by 1% *v*/*v* Triton X-100 for 24 h during the preparation of decellularized human umbilical cord tissue hydrogels [39]. This ultimately resulted in a decellularized human umbilical cord tissue that met DNA standards. In this study, trypsin/EDTA, Triton X-100, and deoxycholic acid were selected as the main reagents for decellularization. With the same processing duration of 5 h, various combinations of single and multiple reagents were explored to determine the optimal decellularization protocol. DNA measurement results showed that the use of trypsin, Triton X-100, and SD individually for 5 h, as well as the sequential use of trypsin and Triton X-100 for 5 h, did not meet the DNA removal criteria. This may be related to the relatively short 5 h treatment time designed for this experiment. If the treatment time for these experimental groups is extended appropriately, it may further reduce the DNA content. Meanwhile, the dsDNA level in the Try + SD group was lower than that in the Try + Tri group, indicating that the ionic detergent SD tends to have a stronger elution effect on the DNA of umbilical cord compared to the non-ionic detergent Tri [38]. The DNA results of the Try + SD group, Tri + SD group, and Try + Tri + SD group initially met the criteria for decellularization. The combined use of ionic detergent SD and non-ionic detergent Triton X-100 tends to have a stronger DNA elution effect on the umbilical cord compared to the individual combinations of trypsin and the two detergents, non-ionic detergent Tri. Specifically, the Try + Tri + SD group exhibited the lowest DNA content (*p* < 0.05), indicating that the combined application of trypsin/EDTA, Triton X-100, and SD can achieve a superior DNA removal effect. Further analysis, coupled with histological analysis, electron microscopy, FTIR, and component determination results, has revealed that sequential treatment with trypsin/EDTA, Triton X-100, and sodium deoxycholate is the most effective method for preserving the natural components of umbilical cord tissue, surpassing the performance of the Try + SD and Try + Tri groups. Although the content of collagen and elastin in the Try + Tri + SD group was lower compared to natural umbilical cord tissue, the difference was not statistically significant. The results of in vitro cytotoxicity experiments preliminarily validated the biocompatibility of this decellularization method. The above findings confirm the effectiveness and advantages of the trypsin/EDTA + TritonX-100+sodium deoxycholate decellularization process in preserving and purifying ECM components, making it a promising tissue engineering decellularized matrix material. The mechanism of action may be as follows: Firstly, trypsin degrades the extracellular matrix and breaks intercellular connections by hydrolyzing the carbon termini of arginine and lysine in non-collagenous proteins at 37 °C, loosening the tissue structure to facilitate the penetration of subsequent chemical reagents. Then, the non-ionic detergent Triton X-100 disrupts DNA–protein interactions and alters cell membrane permeability by breaking lipid–lipid and lipid–protein interactions. Finally, the ionic detergent sodium deoxycholate can more effectively dissolve cells and nuclear membranes, accelerating cell rupture. Throughout this process, physical mechanical shaking provides channels for the penetration of enzymes and chemical reagents, shortening the decellularization time and rapidly destroying cell membranes while minimizing damage to the extracellular matrix. Although the Try + SD and Tri + SD groups were able to meet the DNA removal standards and showed no cytotoxicity in vitro, compared to the Try + Tri + SD group under the same treatment time, these two combinations inflicted greater damage to the ECM and led to a lower content of ECM components.

Previous studies commonly used methods such as histological staining, electron microscopy, and DNA quantitation to assess the decellularization effects on tissues or organs [40,41,42]. In our article, we have employed FTIR detection and added statistical quantification of the FTIR results. Through FTIR detection, we obtained some crucial data. On the one hand, the data visually reflect the trend of changes in the parameters of functional groups on the tissue surface before and after decellularization. On the other hand, the spectral peak areas can be used as a semi-quantitative analysis tool representing the content of specific chemical functional groups of collagen and GAGs. By further analyzing the FTIR results of collagen, we obtained a meaningful finding that the collagen in this material possesses a relatively intact triple-helical structure. Therefore, we believe that FTIR detection can serve as an important tool for characterizing decellularized tissues, which is conducive to further analyzing whether the macromolecular structure of the tissue has changed before and after decellularization. At the same time, our detection results indicate that after decellularization, the peak areas in the Fourier-transform infrared spectrum show an increasing trend. This trend may be attributed to the further purification and exposure of characteristic functional groups following the removal of impurities, which subsequently enhances the functional group signals of the functional protein components on the surface of the decellularized matrix.

The cytotoxic effects of medical devices are typically tested in vitro using the mouse fibroblast cell line L929, according to the PN-EN ISO 10993-5:2009 method [43]. In our research, however, we utilize liver cells to evaluate the in vitro cytotoxicity of acellular matrix materials, primarily due to the functional characteristics of hepatocytes. Hepatocytes are model cells for assessing drug hepatotoxicity, as they exhibit a unique array of metabolic enzymes and pathways that can significantly influence the toxicity profile of a given substance. Although fibroblasts, like L929, are considered standard due to their robustness and ease of culture, they do not necessarily reflect the same sensitivity or metabolic pathways as liver cells. HepaRG cells have gained widespread recognition as valuable preclinical hepatic models, offering a unique platform for studying liver function, disease progression, and drug metabolism prior to clinical trials [44,45]. By utilizing HepaRG cells, our study is able to uncover potential interactions that might remain undetected in other cell types. Other studies have also conducted in vitro cytotoxicity assessments using human liver cells, given the liver’s central role in drug metabolism [46,47]. Furthermore, using liver cells to assess in vitro cytotoxicity lays the foundation for the future clinical application of acellular materials in liver tissue engineering products.

## 5. Conclusions

Based on the decellularization method of human umbilical cord tissue, this paper explores the impact on the extracellular matrix components of human umbilical cord tissue under different reagent treatment conditions. The results indicate that the combined decellularization strategy utilizing trypsin/EDTA, TritonX-100, and sodium deoxycholate can improve the efficiency in preparing decellularized human umbilical cord dECM, achieving decellularization of the umbilical cord tissue within 5 h while preserving the major matrix components. The establishment of this method lays a foundation for the further application of human umbilical cord decellularized matrix in tissue engineering and regenerative medicine.

## Figures and Tables

**Table 1:** Experimental design.

Group	Processing Steps		
	Step 1	Step 2	Step 3
Native	/	/	/
Try	0.025% *w*/*v* trypsin-EDTA, 37 °C, 5 h, 120 rpm	/	/
Tri	5% *v*/*v* Triton X-100, 5 h, 120 rpm	/	/
SD	4% *w*/*v* SD, 5 h, 120 rpm	/	/
Try + Tri	0.025% *w*/*v* trypsin-EDTA, 37 °C, 1.5 h, 120 rpm	5% *v*/*v* Triton X-100, 3.5 h, 120 rpm	/
Try + SD	5% *v*/*v* Triton X-100, 2.5 h, 120 rpm	4% *w*/*v* SD solution, 2.5 h, 120 rpm	/
Tri + SD	5% *v*/*v* Triton X-100, 2.5 h, 120 rpm	4% *w*/*v* SD solution, 2.5 h, 120 rpm	/
Try + Tri + SD	0.025% *w*/*v* trypsin-EDTA, 37 °C, 1.5 h, 120 rpm	5% *v*/*v* Triton X-100, 1.5 h, 120 rpm	4% *w*/*v* SD, 2 h, 120 rpm

## Data Availability

The data presented in this study are available on request from the corresponding author. The data are not publicly available due to privacy.
